# Efficacy of a Brief Blended Cognitive Behavioral Therapy Program for the Treatment of Depression and Anxiety in University Students: Uncontrolled Intervention Study

**DOI:** 10.2196/44742

**Published:** 2023-08-25

**Authors:** Ece Atik, Johannes Stricker, Magnus Schückes, Andre Pittig

**Affiliations:** 1 Translational Psychotherapy Institute of Psychology University of Goettingen Goettingen Germany; 2 Clinical Psychology Research Group Department of Experimental Psychology Heinrich Heine University Düsseldorf Düsseldorf Germany; 3 University of Mannheim Mannheim Germany

**Keywords:** blended cognitive behavioral therapy, bCBT, digital mental health, e–mental health, depression, anxiety disorder, video psychotherapy, mobile phone

## Abstract

**Background:**

Blended cognitive behavioral therapy (bCBT)—the combination of cognitive behavioral therapy and digital mental health applications—has been increasingly used to treat depression and anxiety disorders. As a resource-efficient treatment approach, bCBT appears promising for addressing the growing need for mental health care services, for example, as an early intervention before the chronification of symptoms. However, further research on the efficacy and feasibility of integrated bCBT interventions is needed.

**Objective:**

This study aimed to evaluate the efficacy of a novel bCBT program comprising short (25 min), weekly face-to-face therapy sessions combined with a smartphone-based digital health app for treating mild to moderate symptoms of depression or anxiety.

**Methods:**

This prospective uncontrolled trial comprised 2 measurement points (before and after treatment) and 2 intervention groups. We recruited university students with mild to moderate symptoms of depression or anxiety. On the basis of the primary symptoms, participants were assigned to either a depression intervention group (n=67 completers) or an anxiety intervention group (n=33 completers). Participants in each group received 6 weekly individual psychotherapy sessions via videoconference and completed modules tailored to their respective symptoms in the smartphone-based digital health app.

**Results:**

The depression group displayed medium to large improvements in the symptoms of depression (Cohen *d*=−0.70 to −0.90; *P*<.001). The anxiety group experienced significant improvements in the symptoms of generalized anxiety assessed with the Generalized Anxiety Disorder-7 scale with a large effect size (Cohen *d*=−0.80; *P*<.001) but not in symptoms of anxiety assessed with the Beck Anxiety Inventory (Cohen *d*=−0.35; *P*=.06). In addition, both groups experienced significant improvements in their perceived self-efficacy (Cohen *d*=0.50; *P*<.001 in the depression group and Cohen *d*=0.71; *P*<.001 in the anxiety group) and quality of life related to psychological health (Cohen *d*=0.87; *P*<.001 in the depression group and Cohen *d*=0.40; *P*=.03 in the anxiety group). Work and social adjustment of patients improved significantly in the depression group (Cohen *d*=−0.49; *P*<.001) but not in the anxiety group (Cohen *d*=−0.06; *P*=.72). Patients’ mental health literacy improved in the anxiety group (Cohen *d*=0.45; *P*=.02) but not in the depression group (Cohen *d*=0.21; *P*=.10). Patient satisfaction with the bCBT program and ratings of the usability of the digital app were high in both treatment groups.

**Conclusions:**

This study provides preliminary evidence for the feasibility and efficacy of a novel brief bCBT intervention. The intervention effects were generalized across a broad spectrum of patient-reported outcomes. Hence, the newly developed bCBT intervention appears promising for treating mild to moderate depression and anxiety in young adults.

## Introduction

### Background

Depression and anxiety disorders are widespread [1,2]. Both disorders have severe consequences for those affected, impairing their social and occupational lives and physical health [3-5]. According to the World Health Organization, depression is the most disabling disease in high- and middle-income countries, topping heart and cerebrovascular diseases [6]. Similarly, anxiety disorders are linked to disability, low educational attainment, and low income [7,8].

University students are particularly at risk for developing depression and anxiety disorders [9]. Several studies suggest that up to 30% of university students display elevated and distressing symptoms of depression or anxiety [10-12]. Some groups of students, such as female students [13,14], first-year students [15,16], and health students [10,17], are at an even greater risk for depression or anxiety. Many university students are in a major transitory phase of their life, maturing from adolescence into adulthood [18]. Changing life habits, family dynamics, academic challenges, and concerns about occupational life after graduation contribute to the elevated risk of developing symptoms of depression or anxiety in university students [19,20]. Elevated symptoms of depression and anxiety induce high costs for university students. For example, depression and anxiety are linked to poorer academic success [21-23] and related withdrawals from university [24].

In up to 75% of the cases, both disorders start in mid-20 years of age [25]. Early interventions for depression and anxiety, implemented before the disorders have reached more severe and chronic states, are frequently recommended [26-28]. Thus, interventions targeting mild to moderate symptoms of depression or anxiety in university students constitute a promising approach for easing the burden of mental illness and preventing severe and chronic trajectories of both disorders. As university students are typically tech-savvy [29], using digital treatment tools may be a promising approach for early interventions in this population.

Commonly recommended treatment options for depression and anxiety include psychotherapy and pharmacotherapy [30]. Regarding psychotherapeutic interventions, cognitive behavioral therapy (CBT) is a well-researched and highly effective approach for treating depression and anxiety [31]. In recent years, computerized and internet-based CBT (iCBT) have increasingly been used [32,33]. However, many studies suggest that stand-alone digital treatments without personal contact with a health care provider may suffer from several shortcomings. For example, adherence to digitalized stand-alone treatment is typically low [34], and the rates of premature treatment termination are high [35,36]. iCBT is rarely personalized and often falls short of meeting patients’ individual needs [37]. Thus, its efficacy and effectiveness are limited when delivered in a stand-alone format [38,39]. The problems of stand-alone digital treatments are often also attributed to the lack of psychotherapist contact providing support and guidance throughout the treatment [35,39,40]. Thus, blended CBT (bCBT) programs, combining face-to-face CBT with digital tools, have been increasingly advocated [41,42].

Previous studies support the efficacy and feasibility of bCBT programs for treating depression and anxiety [43-45]. Some of these studies found stronger symptom reduction in patients receiving bCBT compared with CBT [46-48]. Other studies have compared bCBT programs with CBT programs that comprised more or longer face-to-face sessions than bCBT programs [44,49-51]. Importantly, in these studies, the reduced face-to-face time in bCBT compared with CBT did not lead to the reduced efficacy of bCBT programs compared with more extensive CBT programs [44,49-51]. As resources for mental health care are scarce, even in high-income countries [52], bCBT may be an effective and efficient alternative to face-to-face CBT only. In particular, as an early intervention for mild to moderate symptoms of depression and anxiety in young adults, bCBT may represent a promising treatment option. However, there is little evidence on the efficacy and acceptability of bCBT in this domain. Furthermore, bCBT has seldom been investigated in the vulnerable population of university students.

Further arguments for the use of bCBT can be found in the acceptability of bCBT treatments. Acceptance ratings by psychotherapists and patients have been found to be higher for bCBT than for stand-alone iCBT programs [53,54]. For example, a recent study investigating the attitudes of licensed psychotherapists in Germany found that most practitioners prefer bCBT over stand-alone iCBT programs [53]. During our study period (late 2021 and early 2022), the COVID-19 pandemic caused increased rates of mental disorders and psychological distress in Germany [55,56], further straining the mental health care system and leading to a growing need for mental health services worldwide [57,58]. As the prevalence rates of depression and anxiety have increased over the last few years, also outside of pandemic phases [19,59], the need for effective and resource-efficient programs, such as bCBT, is becoming increasingly pressing.

### Objective

This study aimed to evaluate the efficacy and feasibility of a novel smartphone-based digital health app, *elona therapy*, for use in bCBT. This app was designed to overcome the limitations of iCBT and the existing bCBT programs. Previous bCBT interventions typically use face-to-face sessions and a smartphone-based digital health app as distinct therapeutic elements [46,60]. In contrast, *elona therapy* offers an integrated synthesis of digital and face-to-face elements where therapists can adapt the individually relevant therapeutic content that patients can access on their smartphones. Specifically, digital content incorporated into the treatment is customized by the therapist according to the individual symptoms, personal needs of the patient, and the current psychotherapeutic focus. University students with either mild to moderate symptoms of depression or anxiety received a 6-week bCBT intervention, including face-to-face individual CBT sessions (comprising six 25-min sessions) via videoconference combined with the depression or anxiety module of *elona therapy*. Despite the briefness of the face-to-face sessions, we expected that using the bCBT program with *elona therapy* is associated with significant improvements in mental health outcomes.

## Methods

### Study Design

This study evaluated the efficacy and feasibility of 2 novel bCBT programs, one targeting mild to moderate symptoms of depression and one targeting mild to moderate symptoms of anxiety. We conducted a 6-week trial comprising 2 assessment points (pretreatment [T0] and posttreatment [T1]) and 2 intervention groups (depression and anxiety). During the 6-week intervention, participants received weekly individual CBT sessions (25 min each) over the web via videoconference and used the depression or anxiety module of *elona therapy* on their smartphones. At both assessment points, participants completed a broad set of self-report measures capturing symptoms of depression, symptoms of anxiety, daily functioning level, competencies, and satisfaction with the treatment. All participants provided their informed consent for study participation and data processing for research purposes before the study.

### Ethics Approval

Ethics approval for this study was obtained from the Ethical Board of the University of Mannheim (EK Mannheim 27/2021).

### Participants

**Figure 1:**
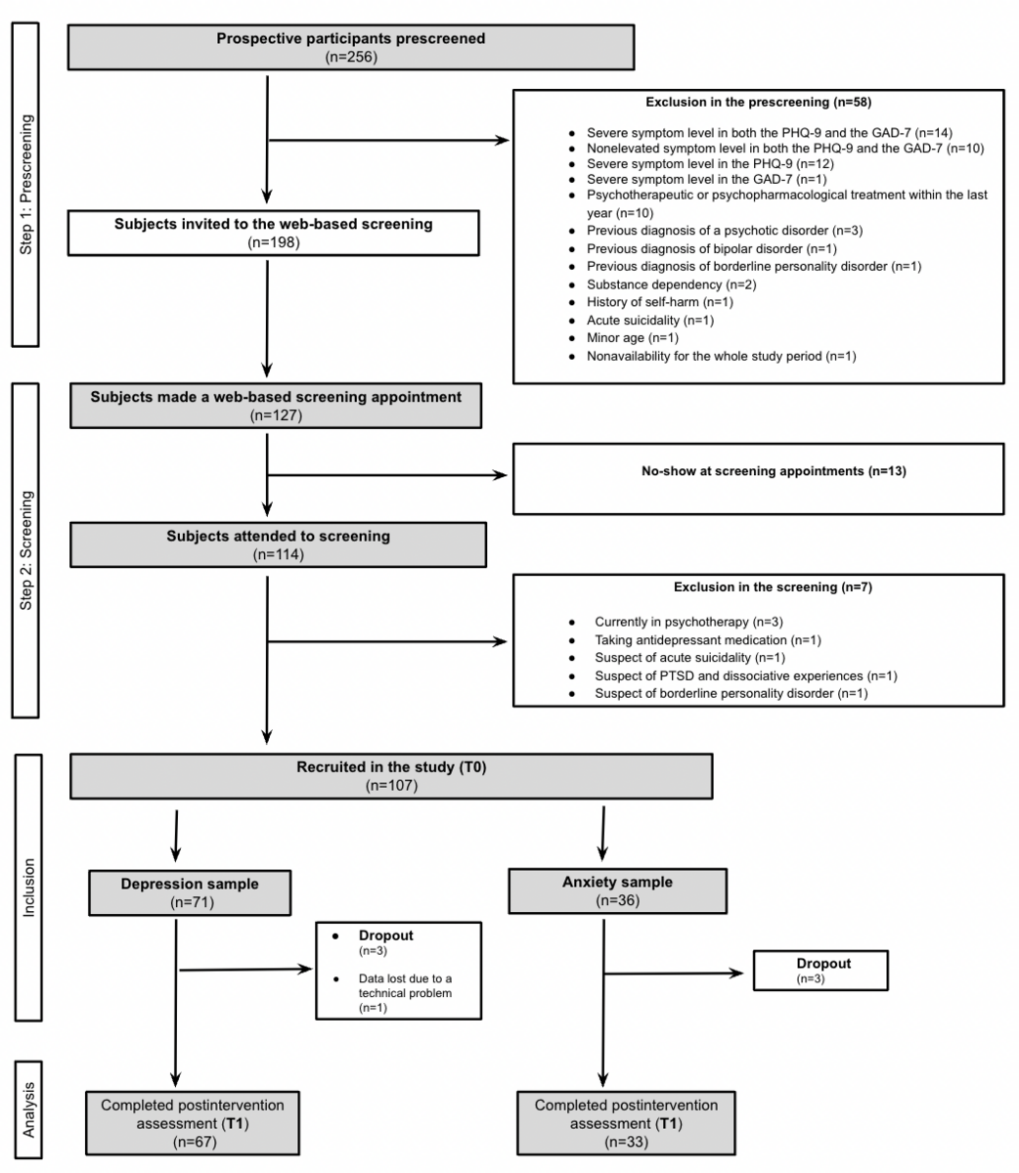
Study flowchart. GAD-7: Generalized Anxiety Disorder-7; PHQ-9: Patient Health Questionnaire-9; PTSD: posttraumatic stress disorder.

This study comprised 2 university student samples, one with mild to moderate symptoms of depression and one with mild to moderate symptoms of anxiety. A total of 107 participants were initially recruited in the study. Depending on their symptomatology, 71 participants were assigned to the depression group and 36 participants were assigned to the anxiety intervention group. Between the pre- and postintervention points, 3 participants dropped out from the depression sample and 3 participants dropped out from the anxiety sample. The data of 1 participant from the depression sample were lost because of a technical problem. In the depression sample, 67 participants (58/67, 87% female; 9/67, 13% male; age: mean 23.61, SD 3.72; range 18-36 years) completed the study by taking part in both assessment points. In the anxiety sample, 33 participants (30/33, 91% female; 3/33, 9% male; age: mean 24.7, SD 3.5; range 19-38 years) completed the study by taking part in both assessment points. Figure 1 displays the study flowchart. Table 1 shows the demographic characteristics of study completers.

Post hoc power analyses (Cronbach α=.05) showed that the study had a high power to detect a large (99.99% for Cohen *d*=0.80), a high power to detect a medium (98.09% for Cohen *d*=0.50), and a small power to detect a small (36.46% for Cohen *d*=0.20) within-group effect in the depression group. Similarly, post hoc power analyses (α=.05) showed that the study had a high power to detect a large (99.37% for Cohen *d*=0.8), a moderate power to detect a medium (79.54% for Cohen *d*=0.5), and a small power to detect a small (20% for Cohen *d*=0.2) within-group effect in the anxiety group.

**Table 1:** Sample characteristics (n=67).

Characteristics		Depression sample (n=67)	Anxiety sample (n=33)
Age (years), mean (SD)		23.61 (3.72)	24.70 (3.50)
Female, n (%)		58 (87)	30 (91)
**University major, n (%)**			
	Sports	1 (1)	0 (0)
	Psychology	11 (16)	4 (12)
	Medicine and related fields (eg, dentistry and pharmacy)	13 (19)	5 (15)
	Social sciences and humanities	22 (33)	15 (45)
	Science and engineering	12 (18)	3 (9)
	Management	3 (4)	4 (12)
	Recent graduates (nonuniversity students)	5 (7)	2 (6)

### Recruitment

University students with mild to moderate symptoms of depression or anxiety were recruited from October 2021 to February 2022 through flyers distributed on campuses and student email groups of several universities in North Rhine-Westphalia, Germany. The study comprised a 2-step inclusion process. In the first step (prescreening), participants completed a web-based survey consisting of the Patient Health Questionnaire-9 (PHQ-9) [61,62], the Generalized Anxiety Disorder-7 (GAD-7) scale [63,64], demographic questions, and further questions examining specific exclusion criteria. Participants who met all the inclusion criteria and none of the exclusion criteria addressed in the web-based assessment were invited to the second step (screening) where they were interviewed by a clinical psychologist (Master of Science).

The inclusion criteria used in the study were as follows: (1) mild to moderate symptoms of depression or anxiety (ie, PHQ-9 or GAD-7 scores between 5 and 15 [64,65]), (2) aged between 18 and 65 years, (3) adequate proficiency in German for engaging in psychotherapy and using the *elona therapy* app, and (4) having a smartphone with iOS or Android operating systems and an internet connection. The exclusion criteria used in the study were as follows: (1) acute suicidality (a score of 2 or 3 on the item 9 of the PHQ-9), (2) current or prior International Classification of Diseases, 10th Revision (ICD-10) bipolar disorder, (3) current or prior ICD-10 borderline personality disorder, (4) current or prior ICD-10 psychotic disorder, (5) symptoms of posttraumatic stress disorder or dissociative experiences, (6) current or prior self-harm (addressed only in the screening with a therapist), (7) current or prior ICD-10 alcohol or substance dependency (except nicotine), and (8) psychotherapeutic or psychopharmacological treatment in the previous year.

The second step, the screening process, comprised a video-based interview with a clinical psychologist currently in advanced postgraduate psychotherapeutic training (interview duration: approximately 25 min). The 2-step inclusion process was used to cross-validate self-report responses with clinical assessments and to assess sensitive inclusion criteria (eg, suicidality) thoroughly and empathically. In the screening interview, inclusion and exclusion criteria were revisited by psychologists, and participants with nonelevated or severe symptoms of depression or anxiety using ICD-10 criteria and those presenting suicidality or other exclusion criteria were excluded. Those with severe symptom levels were advised to consult appropriate health care services and were provided with contact information of emergency services. Participants meeting all the inclusion criteria and none of the exclusion criteria after the second screening step were recruited to the study and assigned to either the depression or the anxiety intervention group, depending on their respective symptomatology. For persons who displayed both elevated symptoms of depression and anxiety, assignment to the depression or anxiety group was jointly decided by the participant and interviewing clinical psychologist (shared decision-making). All interviewing clinical psychologists received interview training from an experienced licensed psychotherapist before the study and supervision throughout the recruitment phase.

### bCBT Intervention Program

The participants received bCBT for depression or anxiety over 6 weeks. The weekly 25-minute video-based individual CBT sessions comprised psychoeducation, internet-based therapeutic exercises (eg, creation of a behavioral activation plan and creation of a personal toolbox), and joint reflections on those tasks. Table 2 summarizes the standardized content of the face-to-face sessions in this study. The treatment manual included 18 digital tasks for the depression group and 16 digital tasks for the anxiety group. The face-to-face sessions were developed based on well-established cognitive behavioral treatment manuals for depression and anxiety [66-69]. CBT sessions were provided by clinical psychologists who currently underwent postgraduate psychotherapeutic training, were regularly seeing outpatients, and received supervision by licensed psychotherapists.

**Table 2:** The standardized content of study sessions for persons with increased symptoms of depression or anxiety.

Session	Manual—depression	Manual—anxiety
1	Establishing a therapeutic relationship Psychoeducation on the characteristics of depression regarding cognitive, affective, behavioral, and physical symptoms	Establishing a therapeutic relationship Psychoeducation on the characteristics of anxiety regarding cognitive, affective, behavioral, and physical symptoms
2	Discussion of homework on personal stress factors and vulnerabilities Psychoeducation on the vulnerability-stress model for explaining the development of depression	Discussion of homework on personal stress factors and vulnerabilities Psychoeducation on the vulnerability-stress model for explaining the development of anxiety
3	Joint reflection on homework on the effects of the interaction among cognitions, affect, and behavior Psychoeducation on downward and upward spirals	Joint reflection on homework on individual anxiety-inducing situations for patients Psychoeducation on the vicious circle of anxiety
4	Discussion of activity planning homework Creation of a concrete plan for an enjoyable activity for the week (behavioral activation)	Psychoeducation and joint reflection on anticipatory anxiety and short- and long-term effects of avoidance and safety behaviors
5	Discussion of the patient’s experiences in the previous week’s planned activity Psychoeducation on cognitive distortions in depression, the cognitive triad of negative views by Beck [70] about oneself, the world, and the future	Summary of previously learned relations between safety behavior and the maintenance of anxiety Psychoeducation on the link between thoughts and bodily symptoms of anxiety and possibilities for restructuring one’s catastrophic thoughts
6	Discussion of homework on cognitive restructuring Summary of all points learned in the bCBT^a^ program. The creation of a personalized “toolbox” with previously learned and helpful content, contributing to relapse prevention	Joint reflection on completed activities on the relation between thoughts and physical reactions Psychoeducation on catastrophic thoughts and interactive practice of questioning them and formulating helpful alternative thoughts

^a^bCBT: blended cognitive behavioral therapy.

To improve their progress between the weekly sessions, patients engaged in additional between-session homework using *elona therapy* on their smartphones. *elona therapy* provides patients with relevant interventions, helpful activities, exercises, and psychoeducational resources related to their respective symptoms. The app content can be individualized according to the patient’s needs, for example, based on their diagnosis, therapy progress, and joint therapeutic decisions. *elona therapy* has been developed based on current treatment recommendations and evidence-based CBT interventions [66-69]. The app is designed to strengthen the active participation of patients in outpatient psychotherapy and integrates therapeutic content into their daily lives. *elona therapy* provides a 2-sided interface for patients (smartphone app) and psychotherapists (web application). Through the web application, psychotherapists can track individual progress, adapt resources and interventions displayed in the patient’s app, and assign tasks based on the content of the face-to-face CBT sessions. Psychotherapists can choose from over 400 available content pieces. Through the smartphone app, patients can access psychoeducational content (text, video, or audio materials), complete interactive homework tasks, record their mood in a diary format, write notes or questions relevant to their therapy sessions, and plan or schedule new activities. In addition, participants can create their personal toolkit of preferred content and tasks within the app. Although the intervention program for this study was manualized to some degree, the participating therapists were encouraged to further individualize the digital content in the *elona therapy* app appropriate for the patient’s needs. Patients were encouraged to use the app approximately 25 minutes per week, mirroring a session duration. Table 3 shows the available content in the *elona therapy* app for patients with symptoms of depression or anxiety. The participating clinical psychologists received training on the use and functions of *elona therapy* by a senior licensed psychotherapist before the study.

**Table 3:** Content of the elona therapy app for persons with increased symptoms of depression or anxiety.

Module and chapter		Content
**Depression**		
	About depression	Psychoeducational content and disorder-specific knowledge about psychotherapy, the symptoms of depression, individual symptoms, comorbid conditions, and factors that promote the development and maintenance of depression.
	My behavior	Relationship between activities and depression, behavior analysis, and activity building and planning. Values-based work.
	My thoughts	Relationship between thoughts and depression. Methods of cognitive restructuring and introduction of the concept of detached mindfulness.
	My emotions	Relationship between emotions and depression. Psychoeducational content to perceive emotions in a more differentiated way, to understand them as indications of needs and support in dealing with emotions.
	My relationships	Depression in a social context. Acquisition of social skills and competencies.
	Relapse prevention for depression	Methods of general relapse prevention such as recognizing individual early warning signs and building a toolbox for difficult situations.
**Anxiety**		
	About anxiety	Psychoeducational content and disorder-specific knowledge about psychotherapy, the symptoms of anxiety disorders, individual symptoms, comorbidities, and the vicious cycle of anxiety.
	Emergence and maintenance of anxiety	Psychoeducational content on the development and maintenance conditions of anxiety disorders and individual application of the acquired knowledge.
	Exposure methods	Information on exposure procedures. More specifically, the mechanisms of exposure are taught and the patient is given the opportunity to prepare for exposure interventions.
	Thoughts and anxiety	Support in identifying and dealing with anxiety-related thoughts. Practicing methods to examine and change anxiety-related thoughts.
	Emotions and anxiety	Psychoeducational content on differentiating emotions and interpreting them as indicators of psychological needs. Developing strategies for dealing with emotions.
	ACT^a^ for anxiety	Learning and practicing methods of ACT to deal with symptoms of anxiety
	Relapse prevention for anxiety	Methods of general relapse prevention. Motivating patients to continuously practice what they have learned.
**Relaxation^b^**		
	About relaxation	Psychoeducational content on relaxation techniques, focus on physical processes
	PMR^c^	Mechanisms of action and goals of PMR according to Jacobson and related exercises
	Imagination	Psychoeducation on imagination exercises and related exercises
	Mindfulness and meditation	Psychoeducation on awareness and meditation techniques and related exercises

^a^ACT: Acceptance and Commitment Therapy.

^b^The Relaxation module was available to both intervention groups.

^c^PMR: progressive muscle relaxation.

### Outcome Measures

This study comprised a broad set of outcome measures, covering symptoms of depression and anxiety, perceived functioning, well-being, satisfaction with the treatment program, and competencies [71-75]. We assessed the symptoms of depression and anxiety in both intervention groups as these symptoms regularly co-occur [76,77].

#### Symptoms of Depression

The symptoms of depression were the primary outcome for the depression group and a secondary outcome for the anxiety group. We assessed the symptoms of depression with 2 well-established self-report instruments: the PHQ-9 [61,62] and the Beck Depression Inventory-II (BDI-II) [78,79]. The PHQ-9 assesses the frequency of symptoms of depression over the past 2 weeks with 9 items (eg, feeling tired or having little energy) using a 4-point Likert scale ranging from 0 (“not at all”) to 3 (“nearly every day”). The BDI-II assesses the severity of symptoms of depression over the past 2 weeks with 21 items (eg, loss of interest) using a 4-point Likert scale ranging from 0 (eg, “I have not lost interest in other people or activities”) to 3 (eg, “It’s hard to get interested in anything”). Many studies have demonstrated the reliability and validity of the German PHQ-9 and BDI-II instruments [79-81]. The Cronbach α value in this study was .85 at T0 and .88 at T1 for BDI-II and .70 at T0 and .78 at T1 for PHQ-9.

#### Symptoms of Anxiety

The symptoms of anxiety were the primary outcome for the anxiety group and a secondary outcome for the depression group. We assessed the symptoms of anxiety with 2 well-established self-report instruments: the GAD-7 [63,64] and the Beck Anxiety Inventory (BAI) [82,83]. The GAD-7 contains 7 items assessing the severity of symptoms of generalized anxiety (eg, trouble relaxing) over the past 2 weeks using a 4-point Likert scale ranging from 0 (“not at all”) to 3 (“nearly every day”). The BAI comprises 21 items assessing the severity of common symptoms of anxiety (eg, numbness or tingling) over the past month on a 4-point Likert scale ranging from 0 (“not at all”) to 3 (“severely—it bothered me a lot”). The German GAD-7 and BAI instruments display satisfactory reliability and validity [63,84]. The Cronbach α value in this study was .79 at T0 and .84 at T1 for GAD-7 and .90 at T0 and .91 at T1 for BAI.

#### Quality of Life

Quality of life was assessed with the WHOQOL-BREF scale [85,86]. This standardized 26-item self-report instrument comprises 5 subscales: overall quality of life and general health (2 items), physical health (7 items), psychological health (6 items), quality of life related to social relationships (3 items), and quality of life related to one’s environment (8 items). Participants rated each item on a 5-point Likert scale (eg, 1=“not at all” to 5=“completely”). Previous studies have demonstrated the reliability and validity of the German WHOQOL-BREF [87]. The Cronbach α value in this study was .72 at T0 and .72 at T1 for the overall quality of life and general health domain, .70 at T0 and .77 at T1 for the physical health domain, .78 at T0 and .81 at T1 for the psychological health domain, .47 at T0 and .61 at T1 for the social relationships domain, and .72 at T0 and .73 at T1 for the environment domain.

#### Functioning in Work and Social Life

Patients’ adjustment to work and social life was assessed with the Work and Social Adjustment Scale (WSAS) [88,89]. This instrument captures impairments in relevant life domains beyond the symptom level [88,89]. The WSAS contains 5 items (eg, “Because of my depression/anxiety, my ability to work is impaired”) rated on a 9-point Likert scale ranging from 0 (“not at all impaired”) to 8 (“very severely impaired to the point I can’t work”). Higher scores on this scale indicate higher maladjustment. The German WSAS has been previously shown to be a reliable and valid measurement instrument [88]. The Cronbach α value in this study was .77 at T0 and .82 at T1.

#### Perceived Self-Efficacy

We assessed the participants’ perceived self-efficacy with the General Perceived Self-Efficacy Scale (GSE) [90]. This scale measures beliefs about one’s capacity to cope with new, difficult, or stressful situations. The GSE contains 10 items (eg, “It is easy for me to stick to my aims and accomplish my goals”) on a 4-point Likert scale ranging from 1 *strongly disagree* to 4 *strongly agree*. Previous work demonstrated the reliability and validity of the GSE [91,92]. The Cronbach α value in this study was .88 at T0 and .90 at T1.

#### Mental Health Literacy

We assessed mental health literacy (ie, patients’ knowledge about mental disorders and their treatments) with a 20-item version of the Mental Health Literacy Scale (MHLS) [93,94]. The 20-item MHLS includes items assessing general mental health literacy (eg, “I am confident that I know where to seek information about mental illness”) using a 5-point Likert scale ranging from 1 (“strongly disagree”) to 5 (“strongly agree”). The MHLS items used in our study were the items numbered 16 to 35 in the original 35-item MHLS. The items not used in this study focused on identifying different specific mental disorder diagnoses, which is beyond the scope of this study. The original MHLS demonstrated adequate internal consistency and validity [94]. The Cronbach α value for the MHLS in this study was .83 at T0 and .82 at T1.

#### Satisfaction With Treatment

We assessed patient satisfaction with their treatment at T1 using the German version of the Client Satisfaction Questionnaire (CSQ-8) [95,96]. The CSQ-8 captures patient satisfaction using 8 items (eg, “Have the services you received helped you to deal more effectively with your problems?”) with a 4-point Likert scale ranging from 1 (eg, “No, they seemed to make things worse”) to 4 (eg, “Yes, they helped a great deal”). Higher CSQ-8 scores (range 8-32) indicate higher patient satisfaction. The German CSQ-8 has been extensively validated for assessing patient satisfaction in mental health treatment [96,97]. The Cronbach α value in this study was .93.

#### Usability of the elona therapy App

The usability of the *elona therapy* app was assessed with the German version of the System Usability Scale (SUS) [98,99]. The SUS is a self-report instrument comprising 10 items with a 5-point Likert scale ranging from 1 (“strongly disagree”) to 5 (“strongly agree”). The SUS raw scores are summed and then multiplied by 2.5 to facilitate interpretation. According to the SUS interpretation guidelines, a transformed score >68 indicates an above-average usability [100,101]. The German version of the SUS has shown satisfactory reliability and validity in various studies [99]. The Cronbach α value in this study was .89.

#### Serious Adverse Events

Participating therapists were instructed to report in case their patients experienced any serious adverse events. Serious adverse events include negative events that are harmful or threatening beyond transient distress and are life-threatening or require hospitalization. Self-harm, suicidal ideation, or attempts are potentially serious adverse events.

### Statistical Analyses

We evaluated the changes from before to after the treatment using 2-tailed paired sample *t* tests. Statistical analyses were conducted based on completers, including only participants who have completed at least 1 questionnaire after treatment. In addition, we evaluated our measurement instruments’ reliability (Cronbach α), and we computed the pre-post effect sizes (Cohen *d*) for each outcome. For the depression group, we treated the depression measures (PHQ-9 and BDI-II) as primary outcomes and all other measures as secondary outcomes. For the anxiety group, we treated the anxiety measures (BAI and GAD-7) as primary outcomes and all other measures as secondary outcomes.

## Results

### Engagement With the Intervention

On average, participants in the depression sample completed 21.6 tasks and spent a total of 88.12 minutes using the *elona therapy* app during the intervention period. Participants in the anxiety group completed on average 24.1 tasks and spent 106.9 minutes using the *elona therapy* app during the intervention period. The mean number of completed face-to-face sessions in the depression group was 5.64 (SD 0.64; range 2-6), and the mean number of completed face-to-face sessions in the anxiety group was 5.55 (SD 0.62; range 4-6). Patient engagement with *elona therapy* was high; only 1 patient in the depression group was a minimal user, which is defined as using *elona therapy* for <15 minutes for the entire study period, with only 7 minutes of total use time.

### Depression Group

Table 4 displays the means and SDs for the pre- and postintervention points, the results of the 2-tailed paired samples *t* tests, and the corresponding effect sizes (Cohen *d*) for the depression group. Regarding symptoms of depression (primary outcome), we observed significant improvements in the PHQ-9 score (t_63_=−7.23, *P*<.001; Cohen *d*=−0.90) and the BDI-II score (t_65_=−5.66, *P*<.001; Cohen *d*=−0.70). Regarding the secondary outcomes, the symptoms of anxiety assessed with the GAD-7 (t_60_=−6.71, *P*<.001; Cohen *d*=−0.86) and BAI (t_35_=−4.55, *P*<.001; Cohen *d*=−0.76) and maladjustment assessed with the WSAS (t_58_=−3.74, *P*<.001; Cohen *d*=−0.49) significantly decreased. In addition, perceived self-efficacy assessed with the GSE (t_60_=3.93, *P*<.001; Cohen *d*=0.50) and quality of life assessed with the WHOQOL-BREF (t_61_=6.48, *P*<.001; Cohen *d*=0.82 for overall quality of life and general health; t_61_=6.31, *P*<.001; Cohen *d*=0.80 for physical health; t_61_=6.85, *P*<.001; Cohen *d*=0.87 for psychological health; t_61_=4.27, *P*<.001; Cohen *d*=0.54 for quality of life related to social relationships; and t_63_=4.79, *P*<.001; Cohen *d*=0.61 for quality of life related to one’s environment) significantly improved. One secondary outcome, mental health literacy in the MHLS, did not significantly improve during the intervention period (t_59_=1.66, *P*=.10; Cohen *d*=0.21). At the postintervention point, participants rated the usability of the smartphone-based digital health app for treating depression as high (mean usability score of 74.15 out of 100 on the SUS, corresponding to above-average usability [98]). Participants also rated their satisfaction with the bCBT intervention program as high (mean 26.14 out of 32 on the CSQ-8). There were no serious adverse events reported.

**Table 4:** Means and SDs for the pre- and postintervention points, results of the 2-tailed paired samples *t* tests, corresponding effect sizes (Cohen *d*), and sample size for each comparison in the depression group.

Outcome	Preintervention point, mean (SD)	Postintervention point, mean (SD)	*t* test (*df*)^a^	*P* value	Effect size^b^, Cohen *d*	Sample size, n^c^
Symptoms of depression (PHQ-9^d^)	10.82 (3.72)	6.69 (3.47)	−7.23 (63)	<.001	−0.90	64
Symptoms of depression (BDI-II^e^)	19.40 (7.46)	13.33 (7.54)	−5.66 (65)	<.001	−0.70	66
Symptoms of anxiety (GAD-7^f^)	8.12 (3.83)	5.23 (2.88)	−6.71 (60)	<.001	−0.86	61
Symptoms of anxiety (BAI^g^)	12.83 (8.25)	8.61 (5.99)	−4.55 (35)	<.001	−0.76	36
Perceived self-efficacy (GSE^h^)	25.03 (5.37)	27.07 (5.34)	3.93 (60)	<.001	0.5	61
Work and social adjustment (WSAS^i^)	18.09 (7.68)	14.42 (7.71)	−3.74 (58)	<.001	−0.49	59
Mental health literacy (MHLS^j^)	84.91 (8.10)	85.80 (8.3)	1.66 (59)	.10	0.21	60
Quality of life and general health (WHOQOL-BREF^k^)	6.63 (1.66)	7.65 (1.51)	6.48 (61)	<.001	0.82	62
Physical health (WHOQOL-BREF)	25.10 (3.83)	27.98 (3.62)	6.31 (61)	<.001	0.8	62
Psychological health (WHOQOL-BREF)	17.51 (3.58)	20.26 (3.78)	6.85 (61)	<.001	0.87	62
Quality of life—social (WHOQOL-BREF)	9.85 (2.2)	10.97 (2.27)	4.27 (61)	<.001	0.54	62
Quality of life—environmental (WHOQOL-BREF)	31.34 (4.48)	33.24 (3.85)	4.79 (61)	<.001	0.61	62

^a^The *t* test value associated with the 2-tailed paired sample *t* test assessing pre-post differences.

^b^Effect size=Cohen *d* associated with the respective pre-post difference.

^c^Some participants failed to complete specific postintervention measures, leading to slight deviations in the sample size between the comparisons.

^d^PHQ-9: Patient Health Questionnaire-9 [61,62].

^e^BDI-II: Beck Depression Inventory-II [78,79].

^f^GAD-7: Generalized Anxiety Disorder-7 [63,64].

^g^BAI: Beck Anxiety Inventory [82,83].

^h^GSE: General Self-Efficacy Scale [90].

^i^WSAS: Work and Social Adjustment Scale [88,89].

^j^MHLS: Mental Health Literacy Scale [93,94].

^k^WHOQOL−BREF: World Health Organization Quality of Life Scale (brief version) [85,86].

### Anxiety Group

Table 5 displays the means and SDs for the pre- and postintervention points, the results of the 2-tailed paired samples *t* tests, and the corresponding effect sizes (Cohen *d*) for the anxiety group. Regarding symptoms of anxiety (primary outcome), we observed significant improvements in the GAD-7 score (t_30_=−4.45, *P*<.001; Cohen *d*=−0.80) but not in the BAI score (t_31_=−1.96, *P*=.06; Cohen *d*=−0.35). Regarding the secondary outcomes, the symptoms of depression assessed with the PHQ-9 (t_32_=−4.58, *P*<.001; Cohen *d*=−0.8) and the BDI-II (t_21_=−3.53, *P*=.002; Cohen *d*=−0.75) significantly decreased. In addition, perceived self-efficacy (t_30_=3.97, *P*<.001; Cohen *d*=0.71), mental health literacy (t_28_=2.07, *P*=.048; Cohen *d*=0.38), overall quality of life and general health (t_30_=2.53, *P*=.02; Cohen *d*=0.45), and psychological health (t_30_=2.21, *P*=.03; Cohen *d*=0.40) assessed with the WHOQOL-BREF significantly improved. Maladjustment assessed with the WSAS (t_31_=−0.36, *P*=.72; Cohen *d*=−0.06), physical health (t_30_=1.75, *P*=.09; Cohen *d*=0.31), quality of life related to social relationships (t_30_=0.18, *P*=.86; Cohen *d*=0.03), and quality of life related to one’s environment (t_30_=1.38, *P*=.18; Cohen *d*=0.25) assessed with the WHOQOL-BREF did not significantly improve in the intervention period. At the postintervention point, participants rated the usability of the smartphone-based digital health app for treating symptoms of anxiety as high (mean usability score of 81.68 [out of 100] on the SUS, corresponding to above-average usability [98]). Participants also rated their satisfaction with the bCBT intervention program as high (mean 28.21 out of 32 on the CSQ-8). There were no serious adverse events reported.

**Table 5:** Means and SDs for the pre- and postintervention points, results of the 2-tailed paired samples *t* tests, corresponding effect sizes (Cohen *d*), and sample size for each comparison in the anxiety group.

Outcome	Preintervention point, mean (SD)	Postintervention point, mean (SD)	*t* test (*df*)^a^	*P* value	Effect size^b^, Cohen *d*	Sample size, n^c^
Symptoms of anxiety (GAD-7^d^)	11.33 (3.93)	8.23 (4.91)	−4.45 (30)	<.001	−0.80	31
Symptoms of anxiety (BAI^e^)	21 (10.48)	17.69 (10.47)	−1.96 (31)	.06	−0.35	32
Symptoms of depression (PHQ-9^f^)	10.7 (4.28)	7.3 (4.33)	−4.58 (32)	<.001	−0.80	33
Symptoms of depression (BDI-II^g^)	18.06 (9.92)	12.95 (9.22)	−3.53 (21)	.002	−0.75	22
Perceived self-efficacy (GSE^h^)	24.94 (6.43)	27.19 (5.95)	3.97 (30)	<.001	0.71	31
Work and social adjustment (WSAS^i^)	16.61 (8.21)	16.03 (8.15)	−0.36 (31)	.72	−0.06	32
Mental health literacy (MHLS^j^)	86.45 (8.17)	87.34 (7.18)	2.07 (28)	.048	.38	29
Quality of life and general health (WHOQOL-BREF^k^)	6.7 (1.74)	7.16 (1.66)	2.53 (30)	.02	0.45	31
Physical health (WHOQOL-BREF)	24.79 (4.31)	25.9 (4.77)	1.75 (30)	.09	0.31	31
Psychological health (WHOQOL-BREF)	18.39 (4.59)	19.13 (4.64)	2.21 (30)	.03	0.4	31
Quality of life—social (WHOQOL-BREF)	10.33 (2.29)	10.32 (2.23)	0.18 (30)	.86	0.03	31
Quality of life—environmental (WHOQOL-BREF)	30.09 (4.33)	30.68 (3.78)	1.38 (30)	.18	0.25	31

^a^The *t* test value associated with the 2-tailed paired sample *t* test assessing pre-post differences.

^b^Effect size=Cohen *d* associated with the respective pre-post difference.

^c^Some participants failed to complete some postintervention measures, leading to slight deviations in the sample size between the comparisons.

^d^GAD-7: Generalized Anxiety Disorder-7 [63,64].

^e^BAI: Beck Anxiety Inventory [82,83].

^f^PHQ-9: Patient Health Questionnaire-9 [61,62].

^g^BDI-II: Beck Depression Inventory-II [78,79].

^h^GSE: General Perceived Self-Efficacy Scale [90].

^i^WSAS: Work and Social Adjustment Scale [88,89].

^j^MHLS: Mental Health Literacy Scale [93,94].

^k^WHOQOL-BREF: World Health Organization Quality of Life Scale (brief version) [85,86].

## Discussion

### Principal Findings

This study investigated the efficacy and feasibility of a 6-week bCBT program for mild to moderate depression or anxiety in university students. Overall, participants showed improvements in their primary symptoms, which generalized to other symptom domains, quality of life, and self-efficacy. In addition, participants reported high satisfaction with the intervention program and the *elona therapy* app. The intervention evaluated in this study produced within-group effect sizes that were considerably larger than those observed in waitlist control groups over similar periods [102,103] suggesting that the brief bCBT program may be efficacious.

In addition, a previous meta-analysis on the effectiveness of standard evidence-based psychological treatments in practices revealed similarly large pre-post treatment effect sizes for depression (Cohen *d*=0.87) and anxiety (Cohen *d*=0.88), as observed in our study [104]. Importantly, the studies included in this meta-analysis comprised, on average, more face-to-face time with a therapist [104]. Similarly, the effect sizes obtained in this study were similar to the effect sizes observed in previous, more extensive bCBT programs for patients with depression or anxiety disorders, ranging from 12 to 15 weeks [46,105]. Various factors such as measurement instruments and sample characteristics may influence effect sizes, so treatment effects should only be cautiously compared between studies. However, the current data indicated that compared with previous approaches, the evaluated bCBT program is at least an equally effective and resource-efficient intervention for reducing the symptoms of depression and anxiety in university students.

Regarding within-group differences between intervention outcomes, we observed larger effects for the symptoms of depression or anxiety than for secondary outcomes such as work and social adjustment or self-efficacy. This pattern of findings is in line with a previous meta-analysis [104] and may be explained by the explicit focus of our intervention on symptom reduction. In the anxiety group, significant symptom reduction was observed in the GAD-7 but not in the BAI (*P*=.06). The GAD-7 primarily assesses symptoms of generalized (cognitive) anxiety, whereas the BAI comprises various items capturing physical symptoms of anxiety (eg, heat sensations, accelerated heart rate, and shakiness). Our intervention’s focus on the cognitive aspects of anxiety and psychoeducation rather than in vivo exposure therapy may explain this finding.

Although work and social adjustment improved significantly in the depression group, this was not the case for the anxiety group. This may be because of worse work and social adjustment before the intervention in the individuals with the symptoms of depression compared with those with symptoms of anxiety, as it has been found earlier in the literature [106,107]. Similarly, all domains of quality of life significantly improved in the depression group and only some improved in the anxiety group. Follow-up studies with similar sample sizes across symptom groups (ie, depression or anxiety) are required to understand whether the observed differences may be because of the lower sample size of the anxiety group compared with the depression group in this study. Moreover, the intervention program we offered in the anxiety group appeared significantly effective in improving mental health literacy, whereas an improvement in this domain was not significant in the depression group. It remains unclear whether this is because of different patient characteristics in groups or different content provided for those groups, either within sessions or in the *elona therapy* app (Tables 2 and 3).

Regarding the feasibility of the evaluated program, participants in both study groups rated their satisfaction with the intervention as high. Satisfaction ratings were similar to those provided in the study by Romijn et al [105], who found that participants rated their satisfaction with the bCBT program to be 25.61 (SD 4.21) on average or higher than that reported by Kooistra et al [108], who found the mean satisfaction to be 22.71 (SD 4.82). Notably, these previous bCBT programs are more extensive than those we provided, with the one in the study by Romijn et al [105] lasting 15 weeks and the one in the study by Kooistra et al [108] lasting 10 weeks. Participants also indicated high usability of the *elona therapy* modules, corresponding to acceptable usability according to guidelines [101]. High feasibility was also evident from the remarkably low attrition rates. Approximately 94% of the participants in the depression group and 92% of the participants in the anxiety group completed the 6-week bCBT program as intended. Moreover, the participants’ good engagement with the app during the study period further supports the feasibility of the *elona therapy* app. The blended treatment program introduced in this study appears to be a safe treatment option, as no adverse events were reported in this study.

### Limitations and Future Research Directions

As this study was planned as an uncontrolled trial, several limitations point toward future research directions. First, this study comprised only 2 measurement points (before and after the assessment). Future research with more measurement points is needed to establish the ideal duration of the evaluated bCBT intervention (eg, to establish whether incremental symptom reduction occurs after the sixth week). Further work should also test the stability of the obtained improvements through follow-up assessments. Second, as a feasibility study focusing on within-group improvements, this study did not include control groups. Thus, future research using appropriate active (eg, standard CBT treatment) or passive (eg, waitlist control) groups is needed. In addition, dismantling studies could be useful for evaluating the effects of different elements of the *elona therapy* modules. Third, we only performed a completer analysis in this trial, which may have overestimated the intervention’s benefits. However, given the small number of dropouts in both groups, the differences in completer and intent-to-treat analyses were small. Fourth, this study focused on a convenience sample, including a vulnerable population (ie, university students) with mild to moderate symptoms. The findings may have limited generalizability to populations with different characteristics and to other student samples in different cultures or different regions of Germany. Fifth, we only used patient-reported outcomes in this study. The use of only self-report may provide a limited depiction of patients’ situations owing to patients’ possible inaccurate evaluation of their situation or response biases such as social desirability bias. Further work can complement some of the outcome measures (eg, symptoms of depression and anxiety) by a clinical rating scale such as therapist rating questionnaires.

This trial used only a quantitative study design, which may inform researchers only limitedly about the investigated program. To explore patients’ in-depth experiences with this novel bCBT program, an additional qualitative study was needed. As a follow-up to this trial, we invited all participants who had completed the last face-to-face session with their therapist to participate in a follow-up interview conducted by an interviewer who was not involved in the therapeutic process. The results of this qualitative investigation were published in a separate article [109].

Further work is needed to assess the efficacy and feasibility of the evaluated bCBT program in other groups and individuals with more severe symptomatology.

In summary, this feasibility study provided preliminary evidence based on an uncontrolled pre-post trial design for the efficacy of a novel bCBT program for treating mild to moderate depression and anxiety. Promisingly, substantial symptom improvements were generalized across various outcomes and were accompanied by high patient satisfaction and adherence. Future research is needed to evaluate the efficacy of the evaluated program in different populations and over longer time intervals.

## Abbreviations

BAI: Beck Anxiety Inventory
bCBT: blended cognitive behavioral therapy
BDI-II: Beck Depression Inventory-II
CBT: cognitive behavioral therapy
CSQ-8: Client Satisfaction Questionnaire
GAD-7: Generalized Anxiety Disorder-7
GSE: General Self-Efficacy Scale
iCBT: internet-based cognitive behavioral therapy
ICD-10: International Classification of Diseases, 10th Revision
MHLS: Mental Health Literacy Scale
PHQ-9: Patient Health Questionnaire-9
SUS: System Usability Scale
WSAS: Work and Social Adjustment Scale
